# H1 Antihistamines—Promising Candidates for Repurposing in the Context of the Development of New Therapeutic Approaches to Cancer Treatment

**DOI:** 10.3390/cancers16244253

**Published:** 2024-12-20

**Authors:** Ewa Trybus, Wojciech Trybus

**Affiliations:** Department of Medical Biology, Jan Kochanowski University of Kielce, Uniwersytecka 7, 25-406 Kielce, Poland

**Keywords:** cancer tumor microenvironment, drug repurposing, cancer therapy, cell resistance, histamine, H1-antyhistamines drugs, non-receptor mechanisms, apoptosis, alternative cell death pathways

## Abstract

Despite significant progress in the field of clinical oncology in terms of diagnostic and treatment methods, the results of anticancer therapy are still not fully satisfactory, especially due to limited response and high toxicity. This has forced the need for further research to finding alternative ways to improve success rates in oncological treatment. A good solution to this problem in the context of rapidly obtaining an effective drug that works on multiple levels of cancer and is also safe is the global strategy of repurposing an existing drug. Research into other applications of an existing drug enables a precise assessment of its possible mechanisms of action and, consequently, the broadening of therapeutic indications. This strategy is also supported by the fact that most non-oncological drugs have pleiotropic effects, and most of the diseases for which they were originally intended are multifactorial, which in turn is a very desirable phenomenon due to the heterogeneous and multifaceted biology of cancer. In this review, we will mainly focus on the anticancer potential of H1 antihistamines, especially the new generation that were not originally intended for cancer therapy, to highlight the relevant signaling pathways and discuss the properties of these agents for their judicious use based on the characteristic features of cancer.

## Simple Summary

The repurposing of old drugs has become an alternative strategy for the de novo synthesis of drugs for the treatment of various diseases, including cancer. This review focuses on existing information concerning the antitumor activity of H1 antihistamines, especially new-generation drugs originally intended for anti-allergic therapy, providing new insights into their potential use in oncology. In the context of a multi-targeted approach for the treatment of cancer, attention was given to data on the signaling pathways and cellular mechanisms in which these drugs may be involved. In order to understand the importance of antihistamines as good candidates for repurposing, this article addresses general issues related to the problems of oncological treatment, the desirable characteristics of a potential anticancer drug, and the role of histamine and its receptors, especially the H1R receptor, in the development and progression of cancer.

## 1. Introduction

Cancer is the second most common cause of death worldwide. In 2022, there were an estimated 20 million new cancer cases and 9.7 million deaths. About 1/5 people will develop cancer during their lifetime, about 1/9 men and 1/12 women will die from the disease. It is predicted that by 2050, the number of cancer cases will reach 35 million [https://www.who.int/news, accessed on 18 December 2024]. The prevention and treatment of cancer have therefore become two of the most important public health challenges of the 21st century, also for economic reasons.

Modern cancer treatment involves the use of various methods, such as surgery, radiotherapy, and systemic anticancer therapy, which can be used alone, in combination, or sequentially. The choice of method depends on many factors, including the stage of the disease, resectability, biology, comorbidities, and the overall functional capacity of the patient [1]. The long-term use of chemotherapeutic agents, especially those affecting metabolic pathways and cancer cell signaling, influences the development of tumors and metastases, as well as the degree of treatment response, disease relapse, drug resistance, and cancer stem cells [2]. For this reason, as well as due to the numerous side effects associated with non-selective action against actively proliferating normal cells, and based on a better understanding of tumor biology, “targeted” cancer therapy has been developed with increased specificity for tumor cells and reduced risk of side effects. Targeted therapy includes conventional molecular targeting agents, hormonal agents, immune checkpoint inhibitors, as well as targeted cytotoxic therapy [3,4,5]. Unfortunately, the main drawback of molecularly targeted therapies is the development of drug resistance; therefore, other strategies are being tried to improve the therapeutic efficacy by overcoming such resistance. Currently, many researchers and clinicians are focusing their attention on combination therapies to combat the highly heterogeneous and multifaceted nature of cancer [6,7,8]. The inhibition of a single target in single-agent therapies is often insufficient to achieve the desired therapeutic effect [7]. With the combination therapy, i.e., combining different treatments at the same time, cancers that were previously almost universally fatal have become largely curable [9].

Due to the constantly growing needs, oncology is currently the most active therapeutic area worldwide. Researchers, aiming to provide the highest quality oncological care, are constantly taking action to search for more effective and at the same time safer anticancer drugs with a multidirectional mechanism of action, the effects of which will be long-lasting and will affect many aspects related to the development and progression of cancer.

## 2. Drug Repurposing (DR)—A Strategy to Fight Cancer

Although the increasing technological progress and constantly updated knowledge about the mechanisms of cancer diseases enable the development of new methods of oncological treatment, it takes an average of 10–17 years to translate new forms of therapy into clinical practice, which is not beneficial for the patient due to the need for rapid intervention. After the discovery and design of a potential anticancer compound, subsequent steps take place, such as the analysis of efficacy, toxicity, and the development of a pharmacokinetic and pharmacodynamic profile in in vitro and in vivo experiments [10]. A key step is then to test safety and efficacy on humans in clinical trials, which typically involves three phases. A drug that passes the last of these phases is approved by the relevant drug agencies who, once on the market, monitor its efficacy and collect information on any adverse effects associated with long-term use [11]. Approximately 90% of drugs going through the clinical trial process do not receive approval and do not reach clinical use [7]. In the context of reducing the time to obtain drugs for the treatment of both common and rare diseases, the two main drug regulatory authorities, the FDA (Food and Drug Administration in the United States) and the EMA (European Medicines Agency in Europe), have launched drug repurposing programs for clinically approved drugs [12,13]. By definition, these are activities aimed at identifying new pharmacological/therapeutic uses for old/existing/available drugs or prodrugs previously studied but not approved due to lack of efficacy and safety issues in the originally intended indications [14,15,16]. Other terms for activities related to the development of new applications are “drug repositioning” and “drug reprofiling” [17]. Approximately 30% of these efforts are reportedly successful and ultimately result in the approval of a “repurposed” product and marketing authorization [18]. The advantage of this strategy is that the pharmacokinetics, pharmacodynamics, and toxicity profile of repurposed candidates are well understood, which minimizes the need for validation in a new clinical trial, provided of course that the dose, duration of treatment, and target population are comparable [19,20]. Thus, bypassing several drug development processes reworked at the stage of original destination trials allows compounds/drugs to proceed directly to Phase II clinical trials. However, such a drug also requires post-marketing safety monitoring by the FDA [11,18,21,22]. The added value of drug repurposing is to expand/maximize the therapeutic value of the drug and consequently increase the success rate/achievement of the desired effect compared to the development of new drugs [23]. An agent that has been widely used for years or at least has been in advanced clinical trials without significant side effects is much less likely to fail due to safety issues than a new chemical entity [24]. Typically, the safety profile of a drug in a new indication is similar to that of the original one [25]. However, the risk of failure in later stages of trials (Phase II or III) should always be taken into account, which may be due to insufficient power rather than toxicity [26].

There are two main DR strategies, on-target and off-target. In on-target DR, the known pharmacological mechanism of the drug molecule is applied to a new therapeutic indication: the biological target of the drug molecule is the same but the disease is different [27]. Currently, on-target repurposing accounts for 80–90% of discoveries in this field, as evidenced by the annually published research papers and patent applications [24]. Off-target repurposing, on the other hand, is even more innovative, as it is based on a newly discovered mechanism or one that was known but considered only in the context of the side effects of the drug. Therefore, both the targets and the indications are new [11,28]. To date, untargeted repurposing has not led to significant drug approvals, although there is considerable scientific interest in this direction [24]. The basic assumption of the innovative concept of drug repurposing is that drug candidates exhibit pleiotropic effects beyond their known mechanism of action [29]. Almost all drugs used in the treatment of various human diseases have the potential to affect more than one target [8,19]. This phenomenon has also been confirmed at the transcriptional level by the NIH Library of Integrated Network-Based Cellular Signatures (LINCS) program by profiling changes in gene expression induced by drug treatment [29]. Matching a drug to a disease is determined not only by the drug–target interaction, but also by the possibility of its differential impact on many genes and their products [30,31,32].

The drug repurposing strategy has also become a potential and very attractive way to address the huge needs related to obtaining more effective forms of cancer treatment. The frequent attempts to repurpose drugs in recent years are not only driven by the detailed knowledge of the mechanisms involved in cancer growth and metastasis, but also by the fact that most drugs have off-site targets, which are known anti-cancer targets. More than 2000 drugs have been approved worldwide, each of which has an average of more than six important targets, which in many cases are still not fully understood [26]. Thus, if the targets of candidate anti-cancer drugs are consistent with the complex nature of cancer and overlap to a large extent with the properties of cancer cells that ensure disease development and progression [33], then there is a high probability that these drugs can also benefit cancer patients [10].

In the context of defective apoptosis signaling and multidrug resistance of many cancers [34], great hope is associated with non-oncological drugs with potential properties related to inducing different types of cell death [35], especially inducing lysosomal cell death (LCD). LCD is observed in several physiological and pathological conditions and represents a tool for killing cancer cells [36]. Cancer cells harbor alternative cell death pathways that remain functional even in therapy-resistant cancer cells [37]. In this context, repurposed drugs may therefore represent a promising strategy to facilitate the increased sensitivity of cancer cells to chemo- and radiotherapy [7]. Moreover, they can also be used as prophylactic chemotherapeutic agents for high-risk populations and as adjuvant treatment to prevent relapses [25].

In most cases, candidates for repurposing are drugs originally approved for various non-oncological indications belonging to different therapeutic classes that have demonstrated significant anticancer properties, including cardiovascular drugs [38,39,40], antipsychotics and antidepressants [41,42], antidiabetic drugs [9,43,44,45], antimicrobials [26,46,47], nonsteroidal anti-inflammatory drugs (NSAIDs) [48,49], and immunomodulatory drugs [50,51].

Non-oncological compounds can be used in cancer treatment, such as monotherapy, combination therapy, and multimodal therapy. Drugs targeting cancer features such as maintaining proliferative signaling, blocking cell death, immunosuppression, or dysregulation of cellular energetics may be more effective as monotherapy. In contrast, drugs that have been shown to evade growth suppressors, maintain replicative capacity, induce angiogenesis, activate invasion and metastasis, genomic instability and mutations, and tumor-promoting inflammation are suitable for combination therapy [35,52]. The combination of two or more repurposed drugs consisting of two or more compounds with different mechanisms of action represents an alternative approach to increase therapeutic efficacy [53]. Combination therapies in DR, compared to monotherapy, bring much better effects compared to single-drug treatment, a lower risk of developing drug resistance and the possibility of using much lower drug concentrations, which at the same time translates into a reduction in side effects [54]. In the context of combination therapy to identify effective drugs, Hu’s team developed a computational network biological approach that inhibits the risk of cancer pathway cross-talk, enables filtering and optimizing drug combinations for cancer treatment. Hence, according to the authors, in order to use combination therapy that can significantly improve the efficacy of treatment, it is highly important to identify the most effective drug combinations and interactions, which can accelerate the development of combination therapy [55].

The last aspect is particularly important because the established adverse effect profile for a specific candidate for repurposing concerns doses normally used in routine therapy in the original indications, so in the case of monotherapy there is a risk of toxicity with the use of higher doses of this drug, which may be necessary to achieve effects in oncological therapy [13,56,57]. On the other hand, if the intended use of the repurposed drug is in untested combinations with other drugs, it is advisable to analyze these combinations for potential toxicities and conduct Phase I studies. Regardless of whether studies on an existing drug for repurposing it for anticancer therapy proceed directly to Phase II or have to start from Phase I, they still represent a shorter option for obtaining a potential anticancer agent compared to the time of obtaining the drug de novo [58].

## 3. Inflammation in Cancer

In the treatment of cancer, the clinical outcomes of patients are directly influenced by the heterogeneous and highly dynamic tumor microenvironment (TME—tumor microenvironment) created by the infiltration of innate and adaptive immune cells and the molecular network of many molecules produced and released by tumor cells, stromal cells, and immune cells [59,60]. Due to the mutual interactions between these cells, the processes are driven by positive feedback. Depending on the type of stimulus, the TME can inhibit or promote tumor initiation and progression; therefore, a deeper understanding of tumor immunity is necessary to develop immunotherapeutic strategies [61,62,63,64]. The tumor microenvironment underlies the mechanisms of chronic inflammation, which have a significant impact on cancer by promoting tumor growth and immune evasion. In contrast, acute inflammatory processes involve tumor immune surveillance and antitumor immune responses that can result in tumor regression or elimination [65]. Chronic inflammation can precede or accompany tumor development and promote distant metastasis, contributing to disease progression [7,66]. Chronic inflammation has been implicated in many types of cancer, including breast cancer, lung cancer, colon cancer, pancreatic cancer, and liver cancer [65]. One of the hallmarks of chronic inflammation is the infiltration of inflammatory cells, including macrophages, neutrophils, and various types of lymphocytes [67]. The presence of these cells leads to the continuous production of pro-inflammatory mediators, including the release of cytokines such as TNF-α, interleukin-1 (IL-1) and interleukin-6 (IL-6), and chemokines, which promote cancer cell survival, proliferation, angiogenesis, and invasion [66,68]. In addition, inflammation induces the release of growth factors, including transforming growth factor beta (TGF-β), which promotes tissue remodeling, fibrosis, and angiogenesis, thereby promoting tumor growth. Cancer cells can interact with the tumor immune microenvironment and develop different strategies to avoid T cell destruction [69]. Low or absent expression of adhesion molecules involved in T cell activation and altered expression of immune checkpoint proteins have been noted [70]. H1R-activated macrophages polarize toward an M2-like immunosuppressive phenotype with increased expression of the immune checkpoint VISTA [71]. Prolonged inflammation promotes the accumulation of regulatory T cells (Treg) and suppressor cells, which inhibit immune responses and the cytotoxic activity of effector T lymphocytes (CD8+, known as cytotoxic CTL) and shape the immunosuppressive tumor microenvironment [69]. Tumors with a strong immunosuppressive TME are associated with impaired immune cytotoxicity, are more aggressive, and have a poor prognosis [61]. Immunosuppressive cells can then support abnormal tumor cells to effectively evade immune detection and escape death [72]. The use of inhibitory signaling antagonists and agonists of immune checkpoint co-stimulatory receptors allows the anti-tumor potential of the immune system to be unleashed [73]. 

An important link between inflammation and cancer development is DNA damage, which is caused by reactive oxygen species (ROS) and nitrogen species (RONS) generated in excess by immune cells [74]. The cell’s response to DNA damage is DNA repair (damage repair/response—DDR), cell cycle arrest and apoptosis [75]. In addition, DDR can activate immune-related signaling pathways as a protective mechanism against genome damage, which in turn can exacerbate inflammation, creating a positive feedback loop [76]. RONS are also responsible for enhancing inflammation and disrupting repair mechanisms, leading to mutagenesis and tissue damage [67]. Due to the complex relationships between inflammation, DNA damage, and DNA repair, these processes can easily become deregulated and lead to carcinogenesis [74].

Another important type of inflammation for the development and progression of cancer is inflammation induced by anticancer therapy, including chemo- and radiotherapy [77]. The partial destruction of tumors by therapies and the release of dead cellular material stimulates an inflammatory response generally resembling damage to normal tissue with subsequent repair [78]. In this case, the recognition of dying tumor cells stimulates the production of cytokines and growth factors such as TNF-alpha (tumor necrosis factor α), EGF, IL-6 in the tumor microenvironment, and these in turn can serve as external anti-apoptotic signals (many tumors show a deficiency in apoptotic cell death) or generally can be directed against different types of cell death, which in turn reduces the efficacy of the applied therapy [79,80]. Other cytokines such as IL-22, IL-11, and IL-6 have been implicated in enhancing stem cell phenotypes in cancer, and because these cells are less proliferative and less metabolically active, they thus become less sensitive to many forms of chemo- and radiotherapy. This contributes to drug resistance and relapses after remission in later years [81,82,83].

Tumor cells, by secreting various chemotactic molecules, including stem cell factor (SCF), also recruit mast cells, which accumulate at the periphery of tumors, at the interface with healthy tissues [84]. In fact, mast cells are among the first immune cells recruited to solid tumors. Mast cell density has been described as an indicator of poor prognosis in malignant melanoma, Hodgkin’s lymphoma, cervical and endometrial cancer, esophageal cancer, gastric cancer, breast cancer, lung cancer, colon and prostate cancer, and B-cell chronic lymphocytic leukemia [84,85]. In the tumor microenvironment, mast cells fulfill many important functions beyond their classical participation in Ig-E-dependent allergic reactions. By releasing various pro-inflammatory and anti-inflammatory factors, mast cells can promote neoplastic changes or have anti-tumor effects [86]. The pro-tumor effects of mast cells include interfering with changes in stromal–epithelial interactions, inducing tumor angiogenesis and lymphangiogenesis, and releasing cytokines such as TNF-alpha, interleukin-1, interleukin-6 [87] and growth factors such as vascular endothelial growth factor (VEGF-A, VEGF-B, VEGF-C, VEGF-D), fibroblast growth factor 2 (FGF-2), nerve growth factor (NGF), platelet growth factor-β (PDGF-β) [88,89]. Since mast cells are abundant in the tumor periphery, also in the vicinity of blood vessels, and exhibit high radioresistance and the ability to change the microenvironment, they constitute an important target for tumor immunotherapy [87]. Most importantly, mast cells are the main source of histamine, which can induce tumor cell proliferation via H1R receptors, while inhibiting the immune system via H2R receptors. Mast cells are the main site of histamine production and storage, especially in pathological conditions [90]. Therefore, the use of mast cells and histamine as a target for new therapeutic approaches in cancer seems to be very promising and justified [91].

## 4. The Importance of the Histaminergic System in Carcinogenesis

Previous studies on various cellular and animal models and human clinical trials have provided ample evidence to support the key role of the histaminergic system in the development and spread of cancer and in the response to anticancer therapies. The assessment of the involvement of histamine and its receptors in the progression of cancer creates the opportunity to identify potential molecular targets in the development of new treatment strategies. Histamine receptors are considered to be very promising targets for alternative anticancer treatment [91]. Therefore, great hope is associated with antihistamines that effectively eliminate all histamine-induced symptoms in allergic diseases [92], which also increases the chance of their high efficacy in oncological diseases.

### 4.1. Biological Role of Histamine

Histamine (C_5_H_9_N_3_) is a biogenic, heterocyclic, imidazole monoamine that is formed in the decarboxylation reaction of the biologically inactive amino acid L-histidine by L-histidine decarboxylase (HDC) [93,94]. The enzymatic reaction that produces histamine occurs in the Golgi apparatus, from where the amine is transported and sequestered to secretory granules for storage. In secretory granules, histamine is stored in an ionic bond with acidic residues of the glycosaminoglycan side chains of heparin or in combination with chondroitin sulfate 4 (in mast cells and basophils, respectively). 

In this specific form, cells accumulate histamine in large quantities and release it upon appropriate stimulation [95,96,97]. Histamine can be released during degranulation, which is a consequence of the response to various immunological (immunoglobulin E or cytokines: IL-3, IL-18, IL-33, GM-CSF and SCF) or non-immunological stimuli (calcium ionophore, substance P, opioids) [98]. Immediately after mast cell activation, histamine is rapidly detached from the granule matrix by exchange with sodium ions in the extracellular environment. Histamine can be produced and stored within the same tissue or can be transported to other tissues as a product [99]. Two transporters are responsible for the specific transport of histamine. The first is required for crossing the plasma membrane, and the second, which appears to be the vesicular monoamine transporter 2 (VMAT2), is necessary for crossing the vesicular membrane [94]. VMAT2 gene expression has been found to be modulated by cytokines, positively by TGF-alpha or negatively by IL-1 and TNF-alpha [100].

Histamine exerts a wide range of biological effects by stimulating four types of related, pleiotropic histamine receptors H1R~H4R (H1HR~H4HR, respectively), named according to the order of their discovery and located in a wide variety of tissues. H1R and H2R receptors are widely expressed in contrast to H3R and H4R receptors [101]. The most sensitive to histamine are histamine receptors H3R and H4R, while the activation of H1R and H2R receptors requires much higher concentrations of histamine [102]. All histamine receptors belong to the G protein-coupled membrane receptors (GPCRs). They can be viewed as “cellular switches” whose inactive and active conformations coexist in equilibrium [103]. Histamine receptors transduce stimuli from the external environment across the lipid bilayer to effector sites located within the cell interior [104,105]. Depending on the characteristics of the receptor subtypes with which histamine is paired and the interconnected intracellular signaling pathways, the resulting physiological responses may be different [106]. By binding to the H1R receptor, histamine stimulates smooth muscle contraction in the respiratory and gastrointestinal tract, excites sensory nerves leading to itching and sneezing, and increases vascular permeability via prostacyclin, platelet-activating factor, von Willebrand factor, and nitric oxide (NO) leading to edema. H2R receptors are major mediators of gastric acid secretion and can also enhance mucus production in the respiratory tract and increase vascular permeability [90]. H3R receptors are expressed on histamine-containing neurons and act as presynaptic autoreceptors that mediate feedback inhibition of histamine release and synthesis [94]. H4R expression has been linked to hematopoietic and immunocompetent cells as well as mast cell/eosinophil chemotaxis and recruitment [107,108].

### 4.2. Histidine Decarboxylase Activity and Histamine Concentration in Tumor Tissues and Their Significance for Tumor Progression

The source of histamine is also cancer cells [109]. Numerous studies have shown a very significant participation of histamine in events related to carcinogenesis, such as cell invasion, migration, and angiogenesis [106]. Many cancer cells secrete histamine, which can then modulate the growth of both normal and cancer cells [110]. Similarly to normal cells, histamine is synthesized in cancer cells with the participation of L-histidine decarboxylase [110]. Cancer cells often increase HDC activity, and in some tumors the activity of this enzyme is regulated by histamine itself, which ultimately leads to increased levels of this amine in cancer patients [111,112]. The important role of HDC in cancer is confirmed by the action of its irreversible inhibitor, alpha-fluoromethylhistidine, which inhibits the development of some types of cancer [113]. High levels of HDC and histamine have been associated with gastric, pancreatic and colon cancer [114,115]. The presence of increased HDC expression was considered as a new immunohistochemical marker of neoplastic mast cells [116]. Elevated levels of histamine, with a simultaneously increased expression of HDC and low activity of the enzyme metabolizing exogenous histamine— DAO, were confirmed in human melanoma cell lines HT-168, WM-35, and WM-983 [117,118]. Moreover, according to other reports, increased expression of L-histidine decarboxylase is a significant feature not only of primary melanoma tissue, but also of metastases [111]. Much convincing evidence for the association of high histamine concentration with cancer is provided by the results of numerous clinical studies. Disturbed histamine metabolism and, consequently, its high concentration have been confirmed in the plasma and tissues of ductal carcinomas of the breast. Moreover, it was concluded that higher plasma histamine levels in women with ductal breast cancer did not depend on the size of the tumor, but on the grade of histological malignancy [119].

In turn, other studies have shown significant differences in intracellular histamine metabolism in both benign and malignant tumors, confirmed by high levels of histamine in muscle tissue in malignant tumors [120]. Almost three times higher levels of histamine were detected in whole blood of patients with newly diagnosed solid malignant tumors compared to healthy individuals [121]. Increased HDC activity was also confirmed in non-small cell lung cancer (NSCLC) biopsy samples, and what is more, NSCLC cells have the ability to synthesize, accumulate, and release large amounts of histamine [122,123]. Increased expression of histidine decarboxylase was demonstrated in treatment-resistant cervical cancer cells (HeLa cisR line) and non-small cell lung cancer cells (A549 cisR) [124]. In addition, high histamine levels were associated with a two-fold increase in HDC activity in patients with ovarian, cervical, and endometrial cancer [125]. 

In contrast to the presented data, there are results of studies that did not confirm a positive correlation between HDC activity and histamine concentration in patients with cancer. In the studies of Garcia-Cabarello’s team [120], in which histamine concentration was compared in groups of women with breast cancer and with benign breast tumors, the amine content in malignant tumors was significantly lower despite the simultaneously confirmed increased enzyme activity. Moreover, a significant decrease in histamine concentration may also occur in the blood of cancer patients. Such results were obtained in patients with colon cancer, in whom a correlation between the amount of histamine and the stage of disease advancement was not confirmed [126]. Reduced histamine concentration was also observed in patients with throat and larynx cancer [127]. Moreover, in studies comparing the concentration of histamine in whole blood in patients after radiotherapy and chemotherapy, or in patients with terminal cancer with the concentration of this amine in healthy individuals, the results were very similar or even lower [121]. 

Nevertheless, due to its unique function, L-histidine decarboxylase can serve as a specific marker of histamine biosynthesis, while due to its elevated level in rapidly proliferating cancer cells, it was assumed that it can be an early indicator of cancer. Histidine decarboxylase mRNA levels, histidine decarboxylase protein expression, and enzymatic activity have been shown to be significantly increased in both in vitro studies and in human tumors [128].

### 4.3. Mechanisms of Action of Histamine on Cancer Cells

Cell proliferation and differentiation in different pathophysiological scenarios are the most important processes subject to histamine-induced metabolic reprogramming. Only cells that are able to adapt their metabolic networks to the pressure exerted in such processes can maintain their homeostasis and survive [129]. Cell proliferation is crucial for tumor development and progression, and histamine is the main mediator of this biological process in different types of cancers [10,130]. Histamine can reduce the viability or increase the proliferation of tumor cells by acting as an autocrine growth factor, as some tumor cells can produce histamine, and by exerting paracrine effects between tumor cells and histamine-producing immune cells in the tumor microenvironment [93,115,129]. The bidirectional effects of histamine on tumor growth appear to be dependent on multiple factors, including the concentration of the amine, the type of receptor stimulated in the target cells, and the specificity of the tumor cell line [112,131,132]. In the pancreatic cancer cell line Panc-1, histamine stimulates cell proliferation at nanomolar concentrations, whereas at concentrations above 1 M, histamine inhibits clonogenic growth [133,134]. The amine significantly increases cell proliferation in various breast cancer lines [135], while dose-dependently reducing the growth in human hepatocellular carcinoma cell line HuH-6 [93]. According to other scientific reports, histamine itself can delay the growth and reduce the incidence of induced duodenal and small intestinal tumors, as well as significantly reducing lung metastases [136]. However, due to the demonstrated elevated levels of histamine in various types of rapidly proliferating cancer cells, it has been assumed that histamine may be an early indicator of cancer [128,136]. 

A number of mechanisms leading to the stimulation of tumor cell proliferation have been described for histamine. The effect of histamine on tumor growth is usually dependent on functional histamine receptors expressed directly on the surface of cancer cells or cells surrounding the tumor, and alternatively may be receptor-independent [93,131,134,137]. Both in vitro and in vivo experiments [137,138] have shown that histamine participates in carcinogenesis through interaction with histamine receptors, with the greatest importance attributed to H1R, H2R, H3R receptors, which are responsible for tumor growth, survival, and metastasis [139,140]. It is assumed that histamine receptors may play a role in promoting neoplastic transformation due to the possibility of activating various enzymes [130]. Moreover, numerous studies have shown that histamine receptors are present on cancer cells, which are linked to non-classical transmitter systems, and these in turn may be responsible for the processes of neoplastic transformation and stimulation of cancer cell proliferation [106]. In this context, the pro-tumor effects of histamine have been linked to the Hic receptor, which has been described in microsomes and cell nuclei [141]. Most microsomal Hic sites are located on the cytochromes P450 of all cells, which are involved, among others, in the metabolism of xenobiotics. By interacting with this receptor, histamine is a modulator of cell proliferation, growth, and differentiation, particularly in various melanoma cell lines [101,141]. Such a mechanism is confirmed by the action of compounds such as polyamines, hormones, antihormones, and various antidepressants and antihistamines that block the binding of histamine to P450 [141]. Histamine can induce the proliferation of cervical cancer cells and ovarian cancer cells by affecting the expression of the estrogen receptor [10,142], as well as excessive proliferation of LNCaP cells in hormone-dependent prostate cancer associated with the overexpression of the H3R receptor and its effect on the androgen receptor [134]. Histamine stimulates the growth of human glioma G47 cells by increasing the expression of two proteins that are important for the progression of this cancer: insulin-like growth factors IGF-I and IGF-II [137,143]. 

Histamine has the ability to modulate the synthesis and secretion of cytokines that promote and inhibit the development of cancer in the tumor microenvironment [137,144]. Cytokines participate in all stages of tumorigenesis, acting as the autocrine and paracrine growth and survival factors of these cells. They are important mediators of the immunosuppression typical of cancers. Histamine has been shown to disrupt the balance between Th1/Th2 and Treg in cancer tissues. Histamine changes dendritic cells into Th2 cell promoters, increases the expression of Th2 attractors, and decreases the expression of Th1 effectors, disrupting the cytolytic response [145]. For example, in colon cancer implants, systemic histamine treatment reduces the expression of IFN-γ and IL-12 from Th1, but simultaneously increases the expression of Th2 secreting IL-10 [106]. 

The interaction of histamine with its main receptor HR1 plays a particularly important role in many aspects of cancer development, which explains the great interest in antihistamines in the context of anticancer therapy in recent times [93,146].

## 5. The Importance of the H1R Receptor in Modulating Processes Related to the Development and Progression of Cancer and the Mechanisms of the Anticancer Action of H1 Antihistamines

### 5.1. Histamine H1R Receptor

Compounds that interfere with the action of histamine on H1R receptors are antihistamines that preferentially stabilize the inactive heptahelical conformation of the major histamine receptors via cross-linking, thereby blocking the activation that leads to cell signaling, gene transcription, and specific cellular responses [147]. Due to differences in the chemical structure of these drugs, the site they occupy on the H1R receptor is different from the histamine binding site; hence they are classified as inverse agonists. The action of inverse agonists is to bind to the inactive conformation of the receptor, causing its stabilization, shifting the equilibrium towards the inactive state, which contributes to a decrease in the constitutive activity of the receptor, even in the absence of histamine. For this reason, the term “H1 receptor antagonists” is incorrect and the term “H1–antihistamines” is recommended instead. There are more than 45 H1R antihistamines available worldwide, and all are inverse agonists [103,148,149]. Since the H1R receptor (together with H2R) is widely distributed in the body, it has become the target of virtually all antihistamines in clinical use [101]. H1R receptors are found in a very diverse group of cells, i.e., in nerve cells, smooth muscles of the respiratory tract and vessels, hepatocytes, as well as immune cells such as neutrophils, eosinophils, monocytes, dendritic cells, T and B cells [149,150,151]. The anti-inflammatory/anti-allergic effects of H1R antihistamines mainly depend on their potent histamine inverse agonism, inhibiting even basal histamine H1 receptor signaling [103,152,153].

The association of histamine with the H1R receptor plays an important role in allergic rhinitis, asthma, atopic dermatitis, conjunctivitis, urticaria and anaphylaxis [151], as well as in autoimmune diseases [154].

### 5.2. Generations of Antihistamines

**Figure 1:**
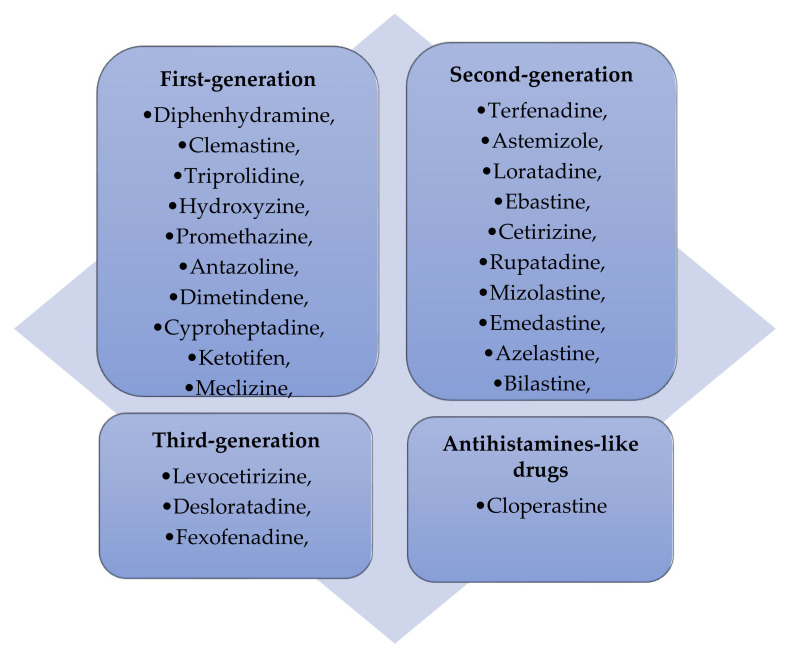
H1 receptor inverse agonists [105,158,159].

Functionally, H1 antihistamines were initially classified (Figure 1) as first-generation drugs, i.e., they readily cross the blood–brain barrier, bind to both central and peripheral histamine receptors, and consequently have potentially sedative and cognitive and psychomotor impairment effects [151]. Major progress in the development of antihistamines came with the introduction of second-generation antihistamines, as these drugs selectively bind to peripheral histamine receptors, thereby posing a lower risk of side effects [155,156,157].

The need to improve both old and new generation preparations has led to the development of newer H1 antihistamine drugs (third-generation), which are metabolites or isomers of second generation drugs. These drugs may be even more effective and have a much lower risk of side effects [149,157,159]. Second- and third-generation H1 antihistamines constitute the basis of the modern therapy for allergic diseases, which, as already mentioned, is due not only to the reduced risk of adverse effects but also to the effective control of inflammatory/allergic conditions and alleviation of accompanying symptoms [92,159,160]. Importantly, newer antihistamines, due to their better risk-to-benefit ratio than first generation antihistamines, seem to be the preferred and appropriate choice in the treatment of children [161,162].

### 5.3. H1R Receptor Function in Cancer

The H1R receptor is highly expressed in many cancer cell lines and tumor tissues [139]. It is expressed in colorectal cancer [163,164,165], bladder cancer [166], ovarian cancer [167,168], breast cancer [169,170,171], liver cancer [68,172], pancreatic cancer [173], lung cancer [174], eye, head and neck cancer [146,172], brain cancer [175], blood cancer [176], skin cancer [109] and soft tissue cancer [154].

As confirmed by previous studies, H1R expression is associated with the prognosis of many types of cancers, including hematological and solid tumors; however, the exact mechanism of the receptor involved in the development of these tumors remains in the sphere of further research [154]. It has been found that the expression of functional H1R receptors promotes cancer progression due to uncontrolled proliferation of cancer cells and tumor growth, which was proven in the case of various lines derived from human breast and epithelial mammary gland cancers (MCF-7, SKBR 3, MDA-453) [177]. Moreover, overexpression of this receptor also contributes to poor prognosis [110,154], which was confirmed in the case of hepatocellular carcinoma (HCC) [68]. The overall data obtained indicate that the major histamine receptor H1R acts as an important oncoprotein and should be a target for potential anticancer therapy [68]. Previous reports clearly indicate H1R antihistamines as excellent candidates for use in anticancer therapy [178].

### 5.4. Mechanisms of the Potential Anticancer Action of H1 Antihistamines

Various mechanisms have been proposed for the potential anticancer effects of H1 antihistamines, and some of them may be completely or partially independent of the histamine H1R receptor [37,166,179,180]. Importantly, H1 antihistamines exhibit multi-directional effects, and most of them represent more than one mechanism relevant for cancer control (Table 1).

#### 5.4.1. Antihistamines in Cancer Immunotherapy

One of the described mechanisms of the anticancer action of H1 antihistamines is the restoration of antitumor immunity, also through checkpoint blockade [189]. Immune checkpoint inhibitors (ICBs) have recently become a novel approach to cancer immunotherapy [194]. Histamine and histamine receptor H1R are often increased in the tumor microenvironment and induce T cell dysfunction and resistance to immunotherapy [106]. In studies assessing the association of tumors with increased expression of the H1R receptor (melanoma, lung cancer, breast cancer, colon cancer) with reduced antitumor immunity in patients treated with immunotherapy, the results clearly confirmed that increased receptor expression was not accompanied by cytotoxic T lymphocyte (CTL) infiltration in the tumor microenvironment. In contrast, fexofenadine hydrochloride restores T cell function inhibited by cancer cell-secreted histamine and improved the response to immunotherapy [71]. In contrast to histamine, fexofenadine promotes macrophages with an M1-like phenotype and increases the activity of CD8+ T cells, alleviating suppression and increasing the response to immunotherapies targeting these lymphocytes [71,191,195]. The action of fexofenadine was associated in this case with a decrease in VISTA (V-domain Ig suppressor of T-cell activation), the expression of which is dependent on the major histamine receptor H1R. Importantly, fexofenadine monotherapy showed similar anti-tumor activity to VISTA antibodies. Moreover, it has been proven that the use of H1 antihistamines during immunotherapy has the property of reversing the effects of cytotoxic lymphocyte checkpoints such as cytotoxic T-lymphocyte-associated antigen 4 (CTLA-4) and programmed death receptors such as programmed cell death protein 1 (PD-1). Fexofenadine inhibits the growth of B16 melanoma tumors that are resistant to anti-PD-1 monotherapy to the same extent as anti-PD-1 and anti-CTLA-4 combination therapy, further demonstrating a critical role for histamine signaling in tumor progression [71,191]. The antitumor response was also investigated in a mouse model of allergic asthma and found that the ongoing allergic response accelerated tumor growth and abolished the host response to immunotherapy, and the use of H1 antihistamines partially reversed these effects [71]. Therefore, it was suggested to use antihistamines in cancer patients with concomitant allergy. The authors of the study clearly emphasized that the major histamine receptor can serve not only as a therapeutic target to enhance the response to immunotherapy, but also as a predictive biomarker of T cell exhaustion and therapeutic efficacy in cancer immunotherapy [71]. The demonstrated mechanism of action of antihistamines is very promising, especially since VISTA (a new member of the B7 family) has become a very promising target for combined cancer immunotherapy due to its well-known role in the regulation of the immune response [194,196,197]. Moreover, despite the success of anti-CTLA-4 and anti-PD-1/PD-L1 therapy, also due to the long-term clinical benefits, the number of people benefiting from ICB is still not satisfactory, as some patients do not respond and some show primary resistance to this type of treatment [198,199]. The effectiveness of cancer immunotherapy has been estimated at only 10–30%, which is why there is a constant effort to improve it and at the same time alleviate the accompanying side effects [71]. Therefore, the discovery of new drugs that have immune checkpoint-blocking activity and the development of rational combination therapies is a way to potential breakthroughs in the development of effective forms of anticancer therapy. In light of the presented data, it has been proposed to use H1 antihistamines as adjuvant agents in combination with immunotherapy in oncology patients with high plasma histamine levels and poorer response to immunotherapy in order to increase the efficacy of anticancer therapy [71,200]. In order to expand knowledge in this direction, it is suggested that randomized, double-blind, placebo-controlled clinical trials should be performed [191]. It is also worth emphasizing that, as assessed in retrospective studies, among forty commonly studied drugs, only H1-specific antihistamines (especially second-generation ones) significantly correlate with better patient survival [71].

In a retrospective study evaluating the effects of CADs, cationic amphiphilic antihistamines, such as desloratadine, cyproheptadine, and ebastine, in patients with lung cancer undergoing immunotherapy compared with non-cationic antihistamines, CADs were associated with an approximately 50% lower risk of mortality and prolonged progression-free survival. Furthermore, these effects were not observed in patients who received CADs prior to immune checkpoint blockade [189].

Importantly, H1 antihistamines can also improve the immune response in neoplastic diseases without the supporting action of immunotherapy. They have been proven to significantly improve survival in people diagnosed with primary invasive cutaneous malignant melanoma (CMM) as well as to reduce the risk of new primary CMM. Hence, their use in the prevention and treatment of melanoma has been suggested [201]. In another study evaluating the potential of loratadine and desloratadine to improve cancer survival through immune checkpoint control, desloratadine was found to be effective in all immunogenic cancer types (stomach, pancreas, colon/rectum, breast, lung, kidney and bladder, prostate, Hodgkin’s lymphoma, and melanoma), but was ineffective in all non-immunogenic cancers analyzed in this study (non-Hodgkin’s lymphoma, brain/central nervous system, thyroid, liver, uterus, ovaries). Loratadine, on the other hand, was associated with improved survival only in ovarian cancer (a non-immunogenic cancer) [145] and some immunogenic cancers, particularly melanoma and lung cancer [145,202]. The better response to desloratadine associated with a significant improvement in women’s survival compared to loratadine and ebastine was also confirmed in another study on H1R-positive breast cancer [190], which was explained by the fact that it is an active metabolite of loratadine with a more potent effect and greater affinity for the main histamine receptor [203].

In relation to loratadine, another mechanism of anticancer action has also been identified. In studies on a colon cancer cell line—CRC, loratadine treatment during irradiation effectively inhibited the growth of the studied cells. According to the presented results, H1R receptor signaling can interfere with the action of ionizing radiation at different levels, increasing DNA damage and activating Chk1 (checkpoint kinase 1 as a predictive biomarker of radiotherapy resistance), thereby inducing cell arrest in the G2/M phase, the most sensitive to radiation [112].

Based on the findings to date, it can be concluded that new-generation H1 antihistamines, such as loratadine and desloratadine, are excellent candidates for repurposing in the context of new anticancer therapy [178], especially since clinical trials have shown almost no significant drug–drug interactions, which is a very desirable feature for drug repurposing strategies [152].

In studies evaluating the anticancer properties of potential drugs for oncological therapy, a very important aspect is the effective inhibition of tumor growth and promotion of apoptosis or other alternative types of cell death. According to numerous data, antihistamines interacting with the H1R receptor effectively inhibit the growth and survival of cancer cells [204,205]. Studies on breast cancer cells with confirmed overexpression of the H1R receptor and at the same time resistant to basic therapy directed at the HER2 receptor (a protein called human epidermal growth factor receptor 2) have shown that terfenadine leads to a decrease in the proliferative capacity of the tested cells and directs them towards the path of cell death [169]. In turn, in an experimental study by the team of de Guadalupe Chávez-López [186], antiproliferative and proapoptotic effects of astemizole were demonstrated in cervical cancer cell lines of different HPV status (CaSki, SiHa, HeLa, INBL and C-33A) with a pronounced expression of Eag1 channels involved in tumor progression and it was suggested that astemizole is an alternative therapeutic option in the treatment of cervical cancer by targeting potassium channels. Similarly, it was proposed that astemizole may have clinical significance in the treatment of prostate cancer, but only in patients with high levels of Eag1 protein, because, at nanomolar concentrations, it significantly reduced cell proliferation only in invasive WPE1-NB26 cells in contrast to the weak expression of the protein in RWPE-1 cells [187]. Both terfenadine and astemizole are old second-generation H1 antihistamines and have been withdrawn in most countries due to very high cardiotoxicity [131,190]. According to the literature data, this toxicity occurs only in cases of overdose significantly exceeding the recommended therapeutic doses [206].

#### 5.4.2. Cationic Amphiphilic Drug-Induction of Lysosomal Cell Death (LMP)

**Figure 2:**
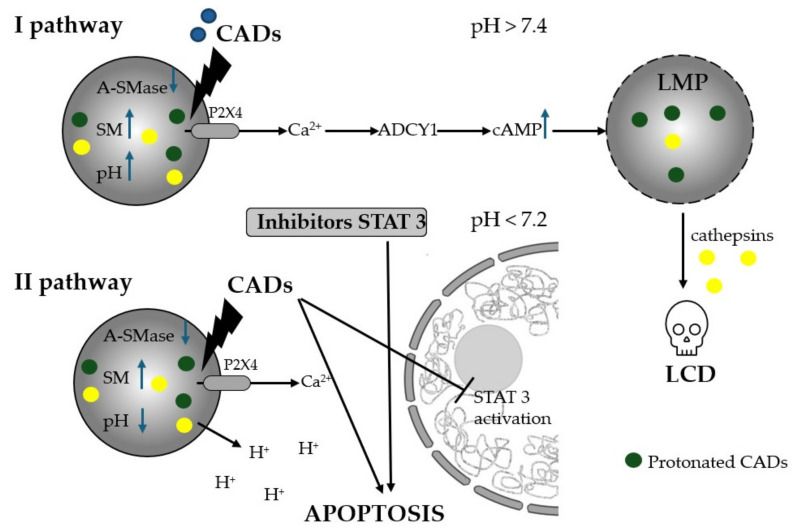
The pathways regulating the CAD-induced cell death pathway [181,182,209]. A-SMase—acid sphingomyelinase, SM—sphingomyelin, P2X4—pH-sensitive purinergic receptor, ADCY1—adenylate cyclase 1, CADs—cationic amphiphilic antihistamines, LMP—lysosomal membrane permeabilization, LCD—lysosomal cell death.

It is worth emphasizing that the positive effect of H1 antihistamines in the context of inducing cell death in cancer cells is not mainly associated with typical antihistamine action. Of particular note are the mechanisms of action of cationic amphiphilic antihistamines (CADs), characterized by a hydrophobic ring structure and a hydrophilic side chain with a cationic amino group. Due to their chemical structure, CADs freely diffuse into acidic lysosomes (Figure 2), where in an acidic environment the basic amino groups of these compounds are protonated, allowing for up to 1000-fold accumulation of the drug in acidic lysosomes [207]. Inside lysosomes, CADs, as weak bases, raise pH and inhibit acid sphingomyelinase (A-SMase, EC 3.1.4.12) and other lysosomal lipases. Subsequently, the accumulation of sphingomyelin (SM) and other lipids acts to destabilize lysosomes in cancer cells, which are inherently fragile and prone to structural changes, leading to increased lysosomal membrane permeability (LMP) [34,37,181,208,209,210,211,212,213,214]. Lysosomal membrane permeabilization, in addition to completely dissipating the lysosomal pH gradient, also allows lysosomal hydrolases to leak into the cytosol, where they function as executors of lysosome-dependent cell death (LCD). Acid sphingomyelinase activity is essential for cancer cells to maintain lysosomal stability and survival, as well as for the multidrug-resistant phenotype [34]. CADs, being effective inhibitors of acid sphingomyelinase, exhibit selective cytotoxicity towards lysosomes of transformed cells [37]. Recently, lysosomes have become an important target for cancer therapy in vitro and in vivo in the context of drug repurposing [215]. CAD-induced inhibition of lysosomal acid sphingomyelinase is necessary, but not sufficient, for subsequent lysosomal membrane permeabilization and cell death. 

Along with the changes in lysosomal lipid catabolism induced by CADs, it has been shown that the early change and response to pH increase is the opening of the lysosomal channel P2RX4 (purinergic receptor P2X4) and the release of lysosomal Ca^2+^ [216,217]. Further studies have shown that CAD-induced and P2RX4-mediated Ca^2+^ release from lysosomes triggers two independent signaling pathways (Figure 2). One of them results in the activation of adenylyl cyclase 1 (ADCY1) and consequently an increase in cytosolic cyclic AMP (cAMP) synthesis [181]. Cytosolic cAMP contributes to the permeabilization of the lysosomal membrane, which causes the leakage of lysosomal hydrolases into the cytosol and the induction of lysosomal cell death [181,218]. The second pathway, which is induced by lysosomal H^+^ efflux, causes an increase in cytosolic acidity that occurs soon after Ca^2+^ release from lysosomes and 1.5–3.5 h before any signs of lysosomal membrane permeabilization are detected. Cytosolic acidification triggers dephosphorylation, lysosomal translocation, and the inactivation of oncogenic signal transducer and activator of transcription 3 (STAT3). The demonstrated mechanism was used to assess the synergistic cytotoxicity of the combined effect of sublethal concentrations of ebastine with WP1066 (STAT3 inhibitor), which did not induce lysosomal membrane permeabilization but influenced the induction of the intrinsic pathway of apoptosis, as indicated by a significant increase in the permeabilization of the outer mitochondrial membrane [182]. 

Moreover, increased intralysosomal pH induced by H^+^ efflux also contributes to the ability of CADs to abolish the phenomenon of multidrug resistance in cancer cells by reducing the entrapment of basic anticancer drugs inside acidic lysosomes [37]. 

Among H1 antihistamines, astemizole, clemastine, ebastine, loratadine, desloratadine, and terfenadine have the property of accumulating in lysosomes and inducing LMP. Moreover, all drugs gave very similar responses in relation to the three non-small cell lung cancer cell lines tested (A549, NCI-H1299, NCI-H661) [37]. In the studies of Petersen’s group, terfenadine showed high cytotoxicity against cancer cell lines of different origins, including ovarian cancer (SKOV3), breast cancer (MCF7), prostate cancer (PC3 and Du145), cervical cancer (HeLa), and bone cancer (U-2-OS). H1 antihistamines such as acid sphingomyelinase inhibitors show high efficacy in various types of cancers, especially those with low activity of this enzyme. However, it has been suggested that monotherapy with these drugs may not be sufficiently effective [34]. This is evidenced by studies evaluating the cytotoxic effects of ebastine and astemizole on human breast adenocarcinoma MCF7 cells when administered separately, in which the tested cells showed significantly reduced sensitivity to these drugs after 6 months of their exposure [181]. Moreover, the resistance phenotype was partially reversed when cells were cultured for 3 months in the absence of CADs. Unfortunately, the mechanism of reduced sensitivity of cancer cells to CADs has not been fully explained [181]. However, considering all the research results obtained in this area and the unique properties of CADs, it is recommended to use them in combination therapy with anticancer drugs to enhance the response of cancer cells, especially those resistant to apoptosis and many drugs. There are numerous examples in the literature confirming such effects. In the studies of Petersen’s team [34], loratadine, astemizole and ebastine administered in sub-micromolar concentrations sensitized non-small cell lung cancer (NSCLC) cells of the NCI-H1299 and NCI-H661 lines to subtoxic concentrations of vinorelbine, thereby reversing multidrug resistance. A detailed analysis of the mechanisms of this combined action showed that the synergistic effect of astemizole and vinorelbine in NCI-H1299 cells resulted from the enhancement of two cell death pathways, both lysosomal and caspase-dependent (the pan-caspase inhibitor z-VAD-fmk inhibited about 60% of cell death induced by vinorelbine alone or in combination with astemizole). Similar sensitization to docetaxel was observed in prostate cancer cells (DU145-MDR line) expressing MDR1 and breast cancer cells (MDA-MB-231-MDR line) treated with low concentrations of CADs [34].

#### 5.4.3. The Effect of Non-CAD on Apoptosis, Proliferation and Cell Cycle of Cancer Cells

Non-CAD drugs also have the ability to sensitize cancer cells resistant to chemotherapeutic agents. It has been proven that the combined use of cisplatin and cloperastine—a drug with H1 antihistamine properties—can potentially provide an effective way to induce apoptosis in human cancers composed of both cisplatin-sensitive cells (HeLa cisR) and naturally occurring cisplatin-resistant cells (HeLa S). As the results from studies show, cloperastine has the ability to kill HeLa cisR cells, because it increases the occurrence of both the early and the late phases of apoptosis, while cisplatin is effective against HeLa S cells. The selective effect of cloperastine has been associated with a molecular mechanism involving the autocrine activity of histamine and high levels of FGF13 (fibroblast growth factor 13) expression [124].

Moreover, the clinical benefit of H1 antihistamines may result from direct cytotoxicity to tumor cells. Loratadine effectively inhibits the growth of tumors derived from human colon cancer cells (COLO 205) in vivo. An in vitro study showed that the antitumor effects of this drug on colon cancer cells were due to cell cycle arrest in the G(2)/M phase and induction of caspase 9-mediated apoptosis. The results of these studies indicated that phosphorylation of Cdc25C by Chk1 (checkpoint kinase 1) plays a major role in the response to G(2)/M arrest by loratadine [183].

Desloratadine promoted apoptosis of bladder cancer cells (EJ and SW780) by modulating the expression of Bcl-2, Bax, and cleaved caspase 3 and 9. Importantly, it simultaneously attenuated the expression of autophagy-related proteins such as Beclin 1, P62, and LC3I/II. Moreover, it dose and time dependently inhibited viability, colony formation ability, and induced cell cycle arrest in the G1 phase. The presented anticancer mechanism of desloratadine also included a strong anti-inflammatory effect by inhibiting the release of interleukin-6 from mast cells and basophils, which significantly reduced the migration and invasion of cancer cells. Moreover, increasing the expression of IL-6 clearly abolished the inhibitory effect of desloratadine on Bcl-2, Bax, Beclin 1, LC3-II/LC3-I in EJ cells [166].

Meclizine has been shown to induce apoptosis in a dose-dependent manner in human colon cancer cell lines, i.e., COLO 205 and HT 29, but its mechanism of action differs from that of loratadine, as it induces cell cycle arrest in the G0/G1 phase, which is associated with increased levels of p53 and p21 proteins and decreased activity of cyclin-dependent kinases 2 and 4 (CDK2 and CDK4, respectively). In contrast, the apoptotic changes induced by meclizine are associated with the upregulation of p53 and downregulation of Bcl-2, thereby causing the release of cytochrome C from mitochondria and translocation of apoptosis-inducing factor (AIF) to the cell nucleus, and consequently activation of caspases 3, 8, and 9 [185].

Another representative of H1 antihistamines, cyproheptadine, reduced the proliferation of HepG2 and Huh-7 HCC cells by blocking cell cycle progression via p38 MAPK activation, and most importantly, demonstrated minimal toxicity to normal hepatocytes [188].

It should be emphasized that the mechanism induced by H1 antihistamines leading to the induction of apoptosis in cancer cells is largely dependent on mitochondria. Dysfunction of these organelles has important consequences for apoptosis, metabolism, and cancer development [192]. In the reports of Fernández-Nogueira [169], terfenadine in vitro inhibited proliferation and activated ERK and p38 MAPK signaling, initiating a mitochondrial apoptotic pathway in cells resistant to HER2-targeted therapy (human epidermal growth factor receptor 2). Receptor HR1 is upregulated in HER2-primary human breast tumors, and HER2 expression correlates with poorer prognosis [112,219]. 

Diphenhydramine has been shown to induce apoptosis in two human acute lymphoblastic leukemia cell lines, CCRF-CEM and Yurkat, which are dependent on Bcl-2 and the mitochondrial pathway. At the same time, it has no cytotoxic effect on PBMCs (peripheral blood mononuclear cells), suggesting that the studied histamine antagonist may have a beneficial therapeutic effect in treatment aimed at the selective killing of cancer cells [220].

In other studies, diphenhydramine, as well as triprolidine, astemizole and terfenadine induced apoptosis in four human melanoma cell lines A375, HT144, HSs294T, MJOI (MJOI showed the lowest sensitivity) with simultaneous selectivity towards normal melanocytes and mouse embryonic fibroblasts. A detailed analysis of the terfenadine mechanism action confirmed that the apoptosis process induced in the studied cells was associated with DNA damage, activation of caspases 2, 3, 6, 8 and 9 (mainly caspase 2) and the mitochondrial pathway. Furthermore, a transient accumulation of treated cells in the S and G2-M phases of the cell cycle before the progression to apoptosis was identified [193]. In other studies, terfenadine-induced apoptosis in A375 melanoma cells was independent of the H1R signaling pathway [180].

Azelastine (azelastine hydrochloride) causes mitochondrial dysfunction in human colon cancer cells (HT29 and DLD-1), as confirmed by the downregulation of Bcl-xL and Bcl-2 protein expression. At the same time, in the same cells, azelastine contributed to the downregulation of p-Drp1 expression, a marker of mitochondrial fission to induce apoptosis. Inhibition of Drp1 activity significantly inhibited the growth and metastasis of cancer cells. Therefore, it was suggested that azelastine could be used as a mitochondrial-targeting agent and apoptosis inducer [192]. In turn, in other studies on HeLa cell lines, azelastine hydrochloride induced changes such as oxidative stress, rough endoplasmic reticulum stress, and mitochondrial dysfunction, which mutually reinforced each other to disrupt cell functions and activate proapoptotic signals. At the same time, the antiproliferative effect of azelastine was demonstrated in the tested cells, confirmed by cell arrest in the S phase and a reduction in the mitotic index [184].

The non-receptor effects of H1 antihistamines are responsible for the anti-inflammatory properties of these drugs, which are essential for effective cancer control, by stabilizing the mast cell membrane and preventing histamine release. This mechanism has been attributed to desloratadine [145]. Moreover, desloratadine also exhibits antioxidant properties by reducing the level of reactive oxygen species (ROS), which are crucial for cancer progression [221,222]. Non-receptor effects may also involve some immune pathways [223,224].

## 6. Conclusions

The presented evidence for the efficacy of commonly used H1 antihistamines in various cancer cell models and in clinical trials confirms the strong anticancer potential of these drugs. In addition to the antihistamine effect, the strength of the analyzed drugs is their ability to induce mechanisms necessary to fight cancer, such as increasing anti-cancer immunity, activating various types of cell death and overcoming the phenomenon of multidrug resistance. Importantly, many studies focus on new-generation drugs that are distinguished by a significantly reduced risk of side effects, which increases their chances of being used in anticancer therapy. Of particular importance are cationic amphiphilic antihistamines (CADs), which show a tendency to inhibit cancer-promoting lysosomal functions and induce lysosomal cell death. Although the mechanisms of action of these drugs have been described, also in relation to combination therapy with other chemotherapeutics, there is still a need to expand research towards finding the strongest combinations of CADs with known chemotherapeutics or compounds with potential anticancer properties. It is also suggested that research should be undertaken to identify signaling pathways and potential drug targets that could be used to promote lysosomal-dependent cell death induced by CADs. The non-receptor action of H1 antihistamines can be seen as an added value, indicating their multidirectional action, which is a highly desirable feature for antihistamine compounds. Both the blocking of the H1R receptor and the non-receptor action of H1 antihistamines are mechanisms of relevance to anticancer therapy. Studies are needed to assess the occurrence of potential side effects or resistance by cancer cells. The data to date indicate important anticancer mechanisms of H1 antihistamines and provide strong arguments for expanding research in this direction.

## Figures and Tables

**Table 1:** Mechanisms of anticancer action of H1 receptor inverse agonists.

ACTION OF H1 ANTIHISTAMINES IN CANCER				
Induction of LMP and Lysosomal Cell Death	Antiproliferative Effect	Increasing Anticancer Immunity. Sensitization to Immunotherapy	Stimulation of Apoptosis	Induction of DNA Damage
Astemizole [37,181,182] Clemastine [37] Ebastine [37,181,182] Loratadine [37] Desloratadine [37] Terfenadine [34,37,182]	Loratadine [183] Desloratadine [166] Azelastine [184] Meclizine [185] Terfenadine [169] Astemizole [186,187] Cyproheptadine [188]	Ebastine [189,190] Loratadine [145,190] Fexofenadine [71,191] Desloratadine [145,189,190] Cyproheptadine [189]	Cloperastine [124] Azelastine [184,192] Desloratadine [166] Terfenadine [169,180,193] Loratadine [183] Meclizine [185] Astemizole [180,186] Triploidine [193] Diphenhydramine [193] Astemizole [193]	Terfenadine [193] Triploidine [193] Astemizole [193] Diphenhydramine [193] Loratadine [112]

LMP—lysosomal membrane permeabilization.
